# Image sharpening algorithms improve clarity of surgical field during 3D heads-up surgery

**DOI:** 10.1186/s40942-023-00462-z

**Published:** 2023-03-30

**Authors:** Kosuke Nakajima, Makoto Inoue, Aya Takahashi, Yuji Yoshikawa, Masaharu Mizuno, Takashi Koto, Tomoka Ishida, Tetsuro Oshika

**Affiliations:** 1 Kyorin Eye Center, Kyorin University School of Medicine, 6-20-2 Shinkawa, Mitaka, Tokyo, 186-8611 Japan; 2 Department of Ophthalmology, Faculty of Medicine, Saitama Medical University, 38, Morohongo, Moroyama, Iruma, 350-0495 Saitama Japan; 3 Department of Ophthalmology, Faculty of Medicine, University of Tsukuba, 1-1-1 Tennoudai, Tsukuba, 305-8575 Japan

**Keywords:** Cataract surgery, Heads-up surgery, Image sharpening algorithm, Three-dimensional surgery, Vitrectomy

## Abstract

**Background:**

Image-sharpening algorithms with color adjustments enable real-time processing of the surgical field with a delay of 4 msec for heads-up surgery using digital three-dimensional displays. The aim of this study was to investigate the usefulness of the algorithms with the Artevo 800^®^ digital microscope.

**Methods:**

Seven vitreoretinal surgeons evaluated the effects of image-sharpening processing on the clarity of the surgical field with the Artevo 800^®^ system that is used for cataract and vitreous surgeries. The scorings were made on a 10-point scale for anterior capsulotomy, phacoemulsification, cortex aspiration, core vitrectomy, and peeling of an epiretinal membrane or an internal limiting membrane. In addition, the images during the internal limiting membrane peeling were processed with or without color adjustments. We also evaluated the skewness (asymmetry in the distribution of the pixels) and kurtosis (sharpness in the distribution of the pixel) of the images to evaluate the contrast with each intensity of image-sharpening.

**Results:**

Our results showed that the mean visibility score increased significantly from 4.9 ± 0.5 at 0% (original image) to 6.6 ± 0.5 at 25% intensity of the image-sharpening algorithm (*P* < 0.01). The visibility scores of the internal limiting membrane increased significantly from 0% (6.8 ± 0.3, no color adjustments) to 50% after the color adjustments (7.4 ± 0.4, *P* = 0.012). The mean skewness decreased significantly from 0.83 ± 2.02 at 0% (original source) to 0.55 ± 1.36 at 25% intensity of the image-sharpening algorithm (*P* = 0.01). The mean kurtosis decreased significantly from 0.93 ± 2.14 at 0% (original image) to 0.60 ± 1.44 at 25% intensity of the image-sharpening algorithm (*P* = 0.02).

**Conclusions:**

We conclude that the image-sharpening algorithms can improve the clarity of the surgical field during 3D heads-up surgery by decreasing the skewness and kurtosis.

**Trial registration:**

This was a prospective clinical study performed at a single academic institution, and the procedures used were approved by the Institutional Review Committee of the Kyorin University School of Medicine (reference number, 1904). The procedures also conformed to the tenets of the Declaration of Helsinki.

## Background

Heads-up surgery (HUS) using digital three-dimensional displays has been introduced for ophthalmic surgeries [1–13]. In HUS, surgeons perform surgical procedures while looking not into the eyepieces of conventional surgical microscopes but at three-dimensional displays placed in front of them displaying images of the surgical field from a high-resolution dual-camera system. Many studies have reported that HUS is a very good option for cataract and vitreoretinal surgeries [1–11]. Eckardt [5] reported that HUS is well suited for vitreoretinal procedures in terms of superior ergonomics and enhancement of the brightness of the surgical field without exposing the retina to excessive light levels. The improved sensitivity of video cameras has enabled surgeons to perform surgeries under low-light conditions. This is another advantage of HUS for vitreous surgery which reduces retinal phototoxicity [14–17]. A comparison of 3D HUS to conventional surgery using a surgical microscope revealed that the outcomes of HUS are equal to or better than those of conventional surgery [3, 4, 8, 13, 17].

Image-sharpening technology is applied in surveillance cameras and is designed to sharpen images in backlighted and nighttime conditions. Hoshi and associates [18] reported that image processing with comb-removal and image-sharpening algorithms improved the visibility of the surgical field during dacryoendoscopy. Tasaki and associates [19] reported that image processing with comb-removal and image-sharpening algorithms improved the visibility of the surgical field significantly during 27-gauge endoscopic vitrectomy. We investigated the usefulness of image-sharpening algorithms during HUS with the Ngenuity^®^ 3D Visualization System [20].

The purpose of this study was to determine whether image-sharpening algorithms can improve the clarity of the surgical field during HUS with the Artevo 800^®^ digital microscope which does not have High Dynamic Range (HDR) video cameras. We also examined whether a color adjustment algorithm can further improve the clarity of the surgical field during HUS.

## Methods

### Subjects

3D HUS was performed during 5 vitreoretinal and 6 cataract surgeries on 6 eyes of 6 patients by a single surgeon (MI). The surgeries were performed with the Artevo 800^®^ (Carl Zeiss Meditec, Oberkochen, Germany) and the medical image enhancer (MIEr^®^, Logic & Design, Tokyo, Japan) at 25% intensity of the image-sharpening algorithm with or without 50% color adjustments.

### Surgical procedures

The Constellation^®^ Vision System (Alcon Laboratories, Fort Worth, TX) was used for the 27-gauge pars plana vitrectomy (PPV) and cataract surgery. The posterior hyaloid cortex was made more visible by an intravitreal injection of triamcinolone acetonide (MaQaid^®^, Wakamoto Pharmaceutical Co., LTD, Tokyo, Japan). Brilliant blue green (BBG) was used to enhance the visibility of the internal limiting membrane (ILM).

A video of the surgery was recorded with the HVO-3300MT^®^ (Sony Group Corporation, Tokyo, Japan) without any image-sharpening. We analyzed the images from the intraoperative videos of the different surgical procedures: anterior capsulotomy with continuous curvilinear capsulorhexis (CCC), lens removal with phacoemulsification and aspiration (PEA), cortex removal with irrigation and aspiration (IA), core vitrectomy (Vit), removal of an epiretinal membrane (ERM), and peeling of the ILM stained with BBG.

To evaluate the effect of the image-sharpening algorithms, the original video images were processed with the medical image enhancer at 25% intensity of the image-sharpening and compared to the original images. The processed values of the image-sharpening intensity ranged from 0 to 255 and the setting of 25% intensity was 64/255 when expressed as a ratio. We analyzed 6 images each during CCC, PEA, and IA, 8 images of core vitrectomy, ERM peeling, and ILM peeling. To evaluate the effect of color adjustments, 9 images of ILM peeling from the same videos after an intravitreal injection of BBG were processed with the medical image enhancer at 50% color adjustments with 25% image-sharpening intensity and compared with no color adjustments at 25% intensity of image-sharpening. The color rate value of the color adjustments ranged from 0 to 64 and the color rate of 16 represented 1.0 (0% color adjustments) and 32 represented 1.5 times (50% color adjustments).

### Visibility scores

Seven vitreoretinal surgeons of intermediate or higher surgical skills (KN, MI, AT, YY, MM, TK, and TI) examined and scored these 43 still images on a scale of 0 to 10 points with 0 as the poorest and 10 as the clearest depending on how clear scorers could see the objects they were manipulating, and how well the objects were contrasted against the background after the information of the patients was masked. The visibility scores were evaluated between the still images with and without 25% intensity of the image-sharpening algorithms. The visibility scores were also evaluated between the still image with and without 50% color adjustments at 25% intensity of the image-sharpening algorithms.

### Skewness and kurtosis

The skewness and kurtosis of those images were calculated using the ImageJ Fiji software [21] to evaluate how the image-sharpening algorithms sharpened the images. The skewness is a measure of asymmetry, i.e., how the distribution of the pixels is pulled to one or the other side of the peak intensity [22]. For instance, if the skewness is negative, the histogram is negatively skewed, and the frequency over the darker intensities is spread wider. When the absolute value of the skewness is near zero, the image is balanced and not extremely dark or bright. Thus, if the skewness of the surgical images is far from zero, the surgeon can perceive the images as being too dark or too bright to do the surgical procedures. The kurtosis of an image is how high and sharp the central peak of the histogram of the brightness of the pixels is [22]. If the kurtosis is high, there are many pixels that have the same pixel value in the image. Thus, the image is of low contrast.

### Statistical analyses

The Wilcoxon signed-rank test was used to compare the two groups. The intra-rater reliability among the examiners was evaluated with the intraclass correlation coefficient (ICC). All statistical analyses were performed using SPSS (version 28.0; IBM, Armonk, New York, NY, USA).

## Results

All surgeries were performed successfully without intraoperative complications with the Artevo 800^®^ and the medical image enhancer with the image-sharpening algorithms at 25% intensity or 25% intensity of image-sharpening with 50% color adjustments during ILM peeling with BBG staining.

### Visibility scores

**Fig. 1:**
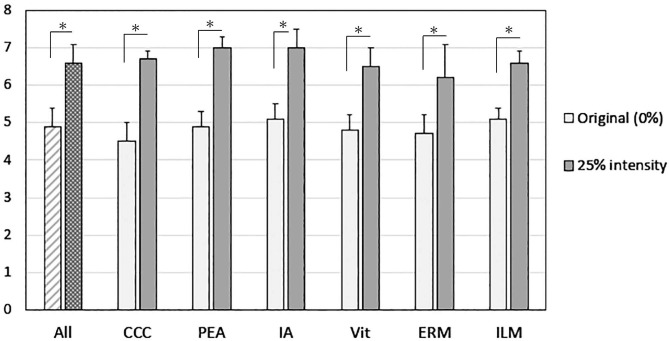
Mean visibility scores for cataract and vitreous surgeries. The mean visibility score of all procedures increases significantly with 25% intensity of the image-sharpening. The visibility scores for CCC, PEA, IA, Vit, and ERM increase significantly with 25% intensity. CCC = continuous circular capsulorhexis, PEA = phacoemulsification and aspiration, IA = irrigation and aspiration, Vit = core vitrectomy, ERM = epiretinal membrane peeling, ILM = internal limiting membrane peeling

The mean visibility score increased significantly from 4.9 ± 0.5 at 0% for the original image to 6.6 ± 0.5 at 25% intensity for the enhanced image (*P* < 0.01, Wilcoxon signed rank test, Fig. 1). The visibility scores for CCC, PEA, IA, Vit, and ERM increased significantly from 0 to 25% intensity of the image-sharpening (CCC, *P* = 0.027; PEA, *P* = 0.027; IA, *P* = 0.028; Vit, *P* < 0.01; ERM, *P* = 0.012; ILM, *P* < 0.01). The visibility score for the ILM with color adjustments increased significantly from 0% (6.8 ± 0.3, no color adjustments) to 50% after the color adjustments (7.4 ± 0.4, *P* = 0.012). The ICC of the visibility scores without the image-sharpening algorithms and with 25% intensity was 0.760 (95% CI: 0.506; 0.943, *P* < 0.001) and 0.802 (95% CI: 0.564–0.955, *P* < 0.001) during CCC, 0.851 (95% CI: 0.650; 0.967, *P* < 0.001), and 0.831 (95% CI: 0.622; 0.962, *P* < 0.001) during PEA, 0.830 (95% CI: 0.606; 0.962, *P* < 0.001), and 0.850 (95% CI: 0.639; 0.967, *P* < 0.001) during IA, 0.765 (95% CI: 0.556; 0.942, *P* < 0.001), and 0.720 (95% CI: 0.496; 0.928, *P* < 0.001) during Vit, 0.726 (95% CI: 0.470; 0.932, *P* < 0.001), and 0.580 (95% CI: 0.300; 0.880, *P* < 0.001) during ERM, 0.868 (95% CI: 0.719; 0.970, *P* < 0.001), and 0.781 (95% CI: 0.578; 0.947, *P* < 0.001) during ILM, respectively. The ICC of the visibility scores without the color adjustments and with 50% color adjustments was 0.692 (95% CI: 0.462; 0.918, *P* < 0.001), and 0.693 (95% CI: 0.463; 0.918, *P* < 0.001), respectively. The intra-rater reliability among the examiners was between moderate agreement (0.41–0.60), substantial agreement (0.61–0.80), and almost perfect agreement (0.81–1.00).

### Image clarity during surgeries

**Fig. 2:**
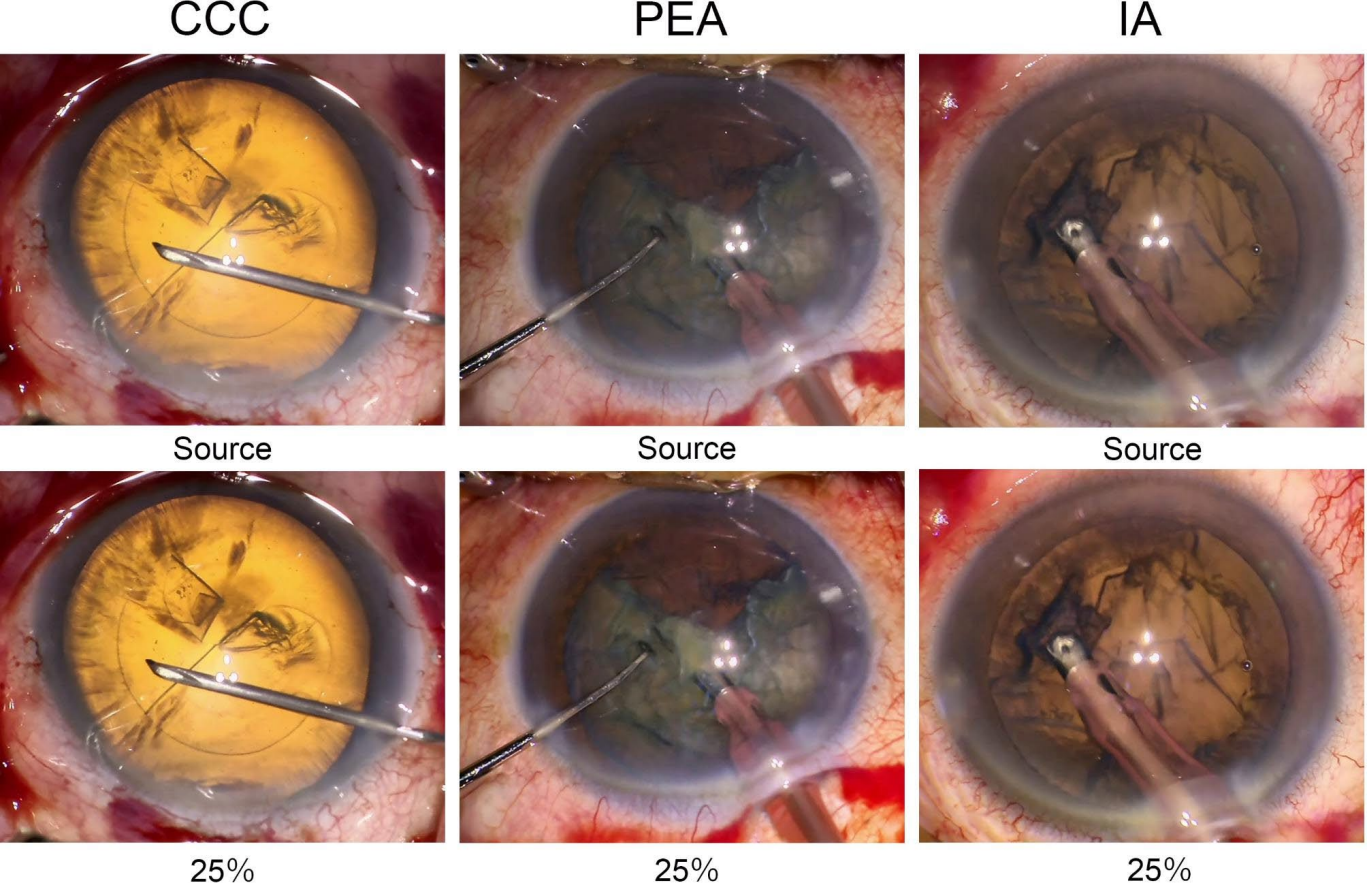
Surgical images of cataract surgery with and without image-sharpening algorithms. The edge of anterior capsulotomy is easily identified during continuous circular capsulorhexis (CCC) with 25% intensity of the image-sharpening. For phacoemulsification and aspiration (PEA) and irrigation and aspiration (IA), the edges of the lens nucleus and the remaining cortex are better highlighted with 25% intensity

**Fig. 3:**
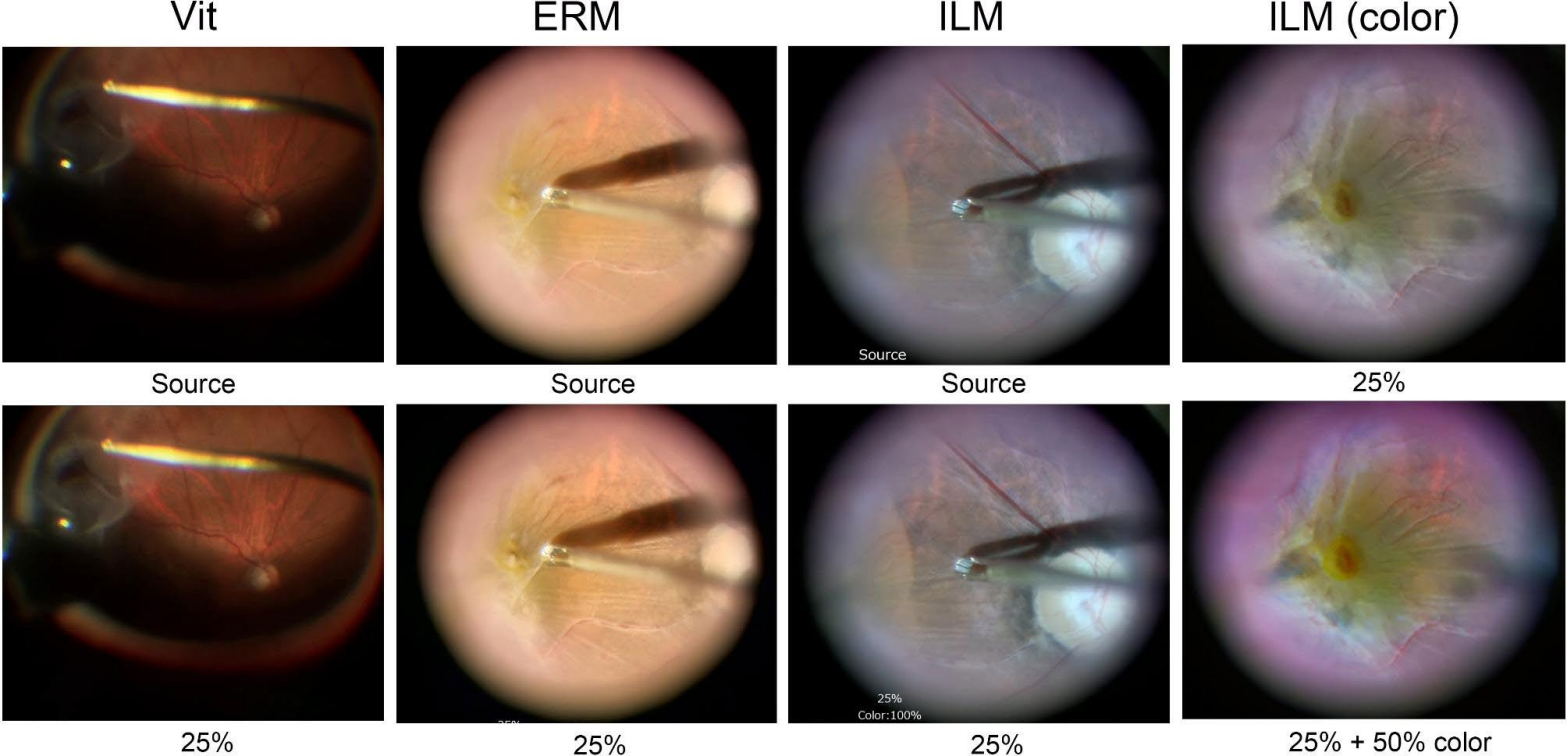
Surgical images of vitreous surgery with and without image-sharpening algorithms. The lightened area of the vitreous cavity is larger and the retinal and choroidal vessels appear more clearly with 25% intensity during vitrectomy. The folds of the epiretinal membrane (ERM) and internal limiting membrane (ILM) stained blue with brilliant blue G appear more clearly with 25% intensity during ERM and ILM peeling. The peeled ILM appears more clearly with the color adjustments that enhance the yellow color of the ILM stained blue with brilliant blue G

For CCC, the edge of the anterior capsule was easier to detect after increasing the intensity of the image sharpening (Fig. 2). For PEA, the edges of the lens nucleus and the remaining cortex were more highlighted with the increase of intensity of image sharpening. For IA, the edge of the anterior capsulotomy and the remaining cortex, and wrinkles of the posterior capsule were seen clearer with increasing intensity of the image-sharpening. For vitrectomy, the area of the vitreous cavity illuminated by a light pipe was larger, and the retinal and choroidal vessels appeared more clearly after increasing the intensity of the image sharpening program (Fig. 3). For ERM peeling, the folds of the ERM appeared clearer with image sharpening, and for ILM peeling, the edges of ILM that were stained blue with BBG appeared more clearly with the image sharpening. For ILM peeling with color adjustments, the peeled ILM appeared more clearly with the color adjustments that was observed the yellow color was emphasized and the color tone was observed clearer with the color adjustments.

### Skewness and kurtosis

To evaluate the contrast of the images, we calculated the skewness and kurtosis of the same images. The mean skewness decreased significantly from 0.83 ± 2.02 at 0% for the original images to 0.55 ± 1.36 at 25% intensity of the image after the image-sharpening algorithm (*P* = 0.01, Table 1). The skewness for PEA, IA, Vit, and ERM decreased significantly when the intensity was changed from 0 to 25% of the image-sharpening algorithm (PEA, *P* = 0.027; IA, *P* = 0.028; Vit, *P* < 0.001; ERM, *P* = 0.012) but the change in the skewness was not significant for CCC, ILM, and ILM with color adjustments.

**Table 1:** Skewness of surgical images

Image-sharpening	All	CCC	PEA	IA	Vit	ERM	ILM	Color adjustment	ILM
Source (0%)	0.83 ± 2.02	0.49 ± 0.23	0.64 ± 0.05	0.65 ± 0.19	3.7 ± 1.7	-1.1 ± 0.8	-0.60 ± 0.42	0% (image-sharpening only)	-0.22 ± 0.52
25%	0.55 ± 1.36	0.40 ± 0.16	0.54 ± 0.03	0.54 ± 0.12	2.5 ± 0.9	-0.77 ± 0.6	-0.52 ± 0.30	50%	-0.20 ± 0.46
*P*-value*	0.01	0.116	0.027	0.028	< 0.001	0.012	0.098	*P*-value*	0.859

(Average ± Standard Deviation) CCC = continuous circular capsulorhexis, PEA = phacoemulsification and aspiration, I/A = irrigation and aspiration, Vit = core vitrectomy, ERM = epiretinal membrane peeling, ILM = internal limiting membrane peeling, * Wilcoxon sign-rank test

The mean kurtosis decreased significantly from 0.93 ± 2.14 at 0% for the original image to 0.60 ± 1.44 at 25% intensity of the image-sharpening algorithm (*P* = 0.02, Table 2). The kurtosis for CCC, PEA, Vit, ERM, and ILM decreased significantly when the intensity was increased from 0 to 25% of the image-sharpening algorithm (CCC, *P* = 0.028; PEA, *P* = 0.027; Vit, *P* < 0.001; ERM, *P* = 0.012; ILM, *P* = 0.014). However, it was not significant for IA and ILM with color adjustments.

**Table 2:** Kurtosis of surgical images

Image-sharpening	All	CCC	PEA	IA	Vit	ERM	ILM	Color adjustment	ILM
Source (0%)	0.93 ± 2.14	-0.04 ± 0.43	-0.35 ± 0.21	0.10 ± 0.78	25.7 ± 22.6	2.4 ± 3.1	0.37 ± 0.84	0% (image-sharpening only)	0.74 ± 1.15
25%	0.60 ± 1.44	-0.27 ± 0.31	-0.51 ± 0.17	-0.15 ± 0.46	8.9 ± 6.9	1.1 ± 2.2	0.04 ± 0.56	50%	0.49 ± 1.22
*P*-value*	0.02	0.028	0.027	0.116	< 0.001	0.012	0.014	*P*-value*	0.859

(Average ± Standard Deviation) CCC = continuous circular capsulorhexis, PEA = phacoemulsification and aspiration, IA = irrigation and aspiration, Vit = core vitrectomy, ERM = epiretinal membrane peeling, ILM = internal limiting membrane peeling, * Wilcoxon sign-rank test

## Discussion

The results showed that the medical image enhancer improved the clarity of the surgical field for CCC, PEA, IA, vitrectomy, ERM peeling, and ILM peeling. In addition, the quantification of the distribution of the pixel intensities for these surgical images showed that the medical image enhancer improved the surgical clarity during cataract and vitrectomy surgeries. The image-sharpening algorithms were tuned to improve the clarity of live images by increasing the dynamic range per pixel that was calculated in real-time by 0.004 Sects. [18, 19]. The scoring by the surgeons showed that the image-sharpening algorithms improved the clarity of the surgical field by increasing the contrast between the object and the background. During cataract surgery, the image-sharpening algorithms showed the edges of the anterior capsulotomy, the wrinkles of the posterior capsule, and the edges of the residual lens cortex more clearly. For vitreoretinal surgery, the image-sharpening algorithms highlighted the vitreous, folds of the ERM, and the border of the areas with ILM that was not peeled and without ILM after it was peeled.

A histogram of the pixel distribution is an important tool for image processing and it gives a graphical representation of the distribution of pixel intensities in a digital image [22]. The skewness and kurtosis are used to evaluate the medical images such as in computed tomography (CT) scans, breast thermographs, and magnetic resonance imaging (MRI) [23–25]. We measured the skewness and kurtosis of the images and compared them with or without the image-sharpening algorithms. The image-sharpening algorithms lowered the skewness into the positive range and brought them closer to zero. This means that the algorithms shifted the bins toward the peak of the histogram [22]. The skewness of surgical images showed that the effectiveness of 25% image sharpening was not too high for procedures using a uniform bright microscope light source. However, for vitrectomy (light guide light source), the enhancement was very effective due to the characteristics of the light from a point source. Also, the image-sharpening algorithms lowered the kurtosis. This means that the algorithms widened and flattened the distribution of the bins of the histogram. The results of the measurements can be interpreted as image-sharpening algorithms that improved the clarity of the surgical images.

The gain and gamma corrections can adjust the overall brightness and darkness uniformly, but they cannot make adjustments when the bright and dark areas are mixed in the same image [26]. Artevo 800^®^ has two integrated 4 K video cameras which are not High Dynamic Range (HDR) video cameras, so the video cameras cannot improve the dynamic range [27, 28]. If the brightness is calculated and set by referring to the histogram of luminance values in a small surrounding range for each pixel unit, the dynamic range can be optimized up to all corners of the screen. In this way, the dark areas are not “blacked out” and bright areas are not “whited out”. The image-sharpening algorithms can be designed to perform this process at high speed and to suppress noise generation and contrast reduction, making it possible to sharpen the images for practical use.

The Ngenuity^®^ 3D Visualization System has dual HDR 2 K video cameras and a color filter system which enables surgeons to adjust the color tone of the surgical image. In contrast, Artevo 800^®^ does not have HDR capability and a color filter system. For these reasons, we experimented with the image-sharpening algorithms for 3D HUS with the Artevo 800^®^. The results showed that the image-sharpening algorithms significantly improved the visual scores for various surgical procedures in simultaneous cataract and vitreous surgery even without HDR capability. The medical image enhancer improved the surgical experience during ILM peeling using the color adjustments by the medical image enhancer even without the color filter function.

This study has limitations. First, we did not assess the quality of the images processed using the medical image enhancer to compare them to the images without the image enhancer during a real-time surgery. Second, we did not assess the visual outcomes on whether the medical image enhancer improved the visual outcomes because we had only a few cases. Further studies are needed to determine whether the proposed algorithm contributes to improved surgical outcomes.

## Conclusions

The image sharpening algorithms with a real-time processing of live images improves the intraoperative clarity during 3D HUS by decreasing skewness and kurtosis. The color adjustments by the medical image enhancer improve the surgical clarity during ILM peeling with BBG staining even without capability of color filter system.

## Data Availability

The data presented in this study are available on request from the first author (K.N.).

## List of abbreviations

HUS: Heads-up surgery
HDR: high dynamic range
PPV: pars plana vitrectomy
BBG: brilliant blue green
ILM: internal limiting membrane
CCC: continuous circular capsulorhexis
PEA: phacoemulsification and aspiration
IA: irrigation and aspiration
ERM: epiretinal membrane
CT: computed tomography
MRI: magnetic resonance imaging
